# Retrospective quality control review of FDG scans in the imaging sub-study of PALETTE EORTC 62072/VEG110727: a randomized, double-blind, placebo-controlled phase III trial

**DOI:** 10.1007/s00259-015-3002-0

**Published:** 2015-02-25

**Authors:** Ivalina Hristova, Ronald Boellaard, Wouter Vogel, Felix Mottaghy, Sandrine Marreaud, Sandra Collette, Patrick Schöffski, Roberta Sanfilippo, Raz Dewji, Winette van der Graaf, Wim J. G. Oyen

**Affiliations:** 1 European Organization for Research and Treatment of Cancer Headquarters, Brussels, Belgium; 2 Department of Radiology and Nuclear Medicine, VU University Medical Centre, Amsterdam, The Netherlands; 3 Department of Nuclear Medicine, The Netherlands Cancer Institute – Antoni van Leeuwenhoek, Amsterdam, The Netherlands; 4 Department of Nuclear Medicine, Maastricht University, Maastricht, The Netherlands; 5 Department of General Medical Oncology, Leuven Cancer Institute, Department of Oncology, University Hospitals Leuven, KU Leuven, Belgium; 6 Istituto Nazionale Tumori, Milano, Italy; 7 GlaxoSmithKline, Oncology R&D, Uxbridge, UK; 8 Department of Medical Oncology, Radboud University Medical Centre, Nijmegen, The Netherlands; 9 Department of Nuclear Medicine, Radboud University Medical Centre, Geert Grooteplein-Zuid 10, 6525 GA Nijmegen, The Netherlands

**Keywords:** Quantitative imaging biomarker, PET/CT, PET, Quality control, Quality assurance, Multicenter clinical study, Standartization, Harmonization, Retrospective review, Guidelines

## Abstract

**Purpose:**

^18^F-Labelled fluorodeoxyglucose (FDG) can detect early changes in tumour metabolism and may be a useful quantitative imaging biomarker (QIB) for prediction of disease stabilization, response and duration of progression-free survival (PFS). Standardization of imaging procedures is a prerequisite, especially in multicentre clinical trials. In this study we reviewed the quality of FDG scans and compliance with the imaging guideline (IG) in a phase III clinical trial.

**Methods:**

Forty-four cancer patients were enroled in an imaging sub-study of a randomized international multicentre trial. FDG scan had to be performed at baseline and 10–14 days after treatment start. The image transmittal forms (ITFs) and Digital Imaging and Communications in Medicine (DICOM) [1] standard headers were analysed for compliance with the IG. Mean liver standardized uptake values (LSUV_mean_) were measured as recommended by positron emission tomography (PET) Response Criteria in Solid Tumors 1.0 (PERCIST) [2].

**Results:**

Of 88 scans, 81 were received (44 patients); 36 were properly anonymized; 77/81 serum glucose values submitted, all but one within the IG. In 35/44 patients both scans were of sufficient visual quality. In 22/70 ITFs the reported UT differed by >1 min from the DICOM headers (max. difference 1 h 4 min). Based on the DICOM, UT compliance for both scans was 31.4 %. LSUV_mean_ was fairly constant for the 11 patients with UT compliance: 2.30 ± 0.33 at baseline and 2.27 ± 0.48 at follow-up (FU). Variability substantially increased for the subjects with unacceptable UT (11 patients): 2.27 ± 1.04 at baseline and 2.18 ± 0.83 at FU.

**Conclusion:**

The high attrition number of patients due to low compliance with the IG compromised the quantitative assessment of the predictive value for early response monitoring. This emphasizes the need for better regulated procedures in imaging departments, which may be achieved by education of involved personnel or efforts towards regulations. LSUV_mean_ could be monitored to assess quality and compliance in an FDG PET/CT study.

## Introduction

Quantitative imaging biomarkers (QIBs) for molecular imaging, such as ^18^F-labelled fluorodeoxyglucose (FDG), have the potential to predict response to therapy early, before changes in tumour size occur. Therefore, molecular imaging could be used for early treatment stratification and modification. Morphological measurements of pathology {e.g. using Response Evaluation Criteria in Solid Tumors (RECIST [3])} have disadvantages in the era of targeted therapy with cytostatic molecules, as tumour size changes do not always occur early. New methods for evaluating response to treatment such as molecular imaging could overcome this challenge. When QIBs become validated endpoints, this has important clinical implications as ineffective treatment can be modified earlier. Several publications have shown the utility of FDG positron emission tomography (PET)/CT for early detection of changes in pathology and correlation with patients’ outcome [4, 5]. The advantages of using biochemical QIBs over morphological in the assessment of cytostatic molecules continues to gain acceptance [6], including patient stratification and prognostic impact before therapy is initiated [7]. However, this requires standardized and reproducible image acquisition, reconstruction and evaluation. Standardization and harmonization of PET/CT in multicentre clinical studies is now well developed after much effort was put into it within the past few years from professional organizations such as the Society of Nuclear Medicine (SNM), European Association of Nuclear Medicine (EANM) and Quantitative Imaging Biomarkers Alliance (QIBA). Despite that, assessment of sites’ compliance with the imaging guideline (IG) in multicentre clinical studies is usually not performed in many trials and only a handful of publications of actual results could be found.

The objective of this study is to explore the need to perform an extensive prospective quality control (QC) on imaging data collected during the course of a study assuring that imaging data actually meet the quality standards needed to address the question at hand. To this end, a retrospective analysis of imaging data collected during a clinical trial was performed.

## Materials and methods

Between October 2008 and February 2010, 369 patients with soft tissue sarcoma whose disease has progressed during or following prior therapy were randomly assigned to receive pazopanib or placebo in the EORTC 62072/VEG110727 phase III clinical trial. The results of the main study were published in 2012 [8]. During this trial patients were offered the opportunity to participate in an FDG PET/CT imaging sub-study. The objective of this imaging sub-study was to investigate whether early changes in tumour metabolism as determined by the maximum standardized uptake value (SUV_max_) were predictive for disease stabilization, response or duration of progression-free survival (PFS). The hypothesis underlying this imaging sub-study (for which 50 patients were planned) was that for a clinically relevant endpoint in patients with a PET response (=25 % decrease in SUV_max_) the PFS was 12 weeks longer than in patients without PET response. The prognostic and predictive value of SUV_max_ on the baseline (BL) and follow-up (FU) FDG PET/CT was to be evaluated independently. As inter-institutional variations in SUV were expected due to scanner and protocol differences, the imaging protocol demanded strict adherence to standardized acquisition and processing parameters. In this retrospective analysis, evaluation of compliance with the IG directly affecting the quantification of PET results (e.g. SUV) and international standards was performed by assessing presence of image artefacts, reviewing the FDG uptake in the liver and verification of scanning-related parameters, such as uptake time (UT), from information available in Digital Imaging and Communications in Medicine (DICOM) headers and image transmittal forms (ITFs).

### Eligibility and recruitment

In order to be eligible for this imaging sub-study, patients had to participate in the EORTC 62072/VEG110727 trial. In addition to the eligibility criteria for the main trial [8], the patients had to meet the following criteria:

- Separate written informed consent for the imaging sub-study.
- At least one tumour lesion ≥2 cm located outside the bone or bladder region.
- Serum glucose ≤200 mg/dl (11 mmol/l). Correction of elevated glucose levels with short-acting insulin was not permitted.
- FU scan if the BL scan showed FDG uptake tumour to background ratio ≥2.
- In case all known metastatic lesions proved to be FDG negative at BL, these patients were not eligible for FU FDG PET and should be replaced.

### Imaging guidelines

A kick-off meeting was organized for all sites and all involved in the study and sub-studies where the IG were discussed to ensure sites could comply. Both PET and PET/CT systems were acceptable. Whole-body FDG had to be performed at two time points: BL (within 2 weeks prior to treatment start) and early FU (approximately 10–14 days after start of treatment).

The proposed acquisition and analysis complied with the National Cancer Institute (NCI) guidelines as given in Shankar et al. [9]:

- Fasting period of a minimum of 4 h prior to FDG administration.
- Prior to imaging, patients were encouraged to drink 700–1,000 ml of water.
- Sedatives such as diazepam were allowed at the discretion of the clinician. However, if this was performed for the BL scan, the amount and timing of the sedative had to be recorded and repeated in the same way for the FU scan.
- Suggested FDG activity 2.5–5.0 MBq/kg, allowed to be individualized depending on the type of scanner and patient habitus per local standard of care.

After intravenous (IV) FDG injection, normal saline was to be used to flush the administration system. The amount of radioactivity in the syringe had to be measured before and after the injection to calculate the net injected dose of FDG, be reported on the ITF and entered accordingly in the scanner. The administration of IV diuretic (e.g. furosemide 10–20 mg) was allowed and had to be given 10–15 min after tracer administration. The patient was to void immediately before scan start.

Given the major impact of UT on SUV [9], the UT was required to be 60 min with maximum acceptable deviation of ± 5 min. The UT of the BL scan had to be recorded and the same UT had to be used for the FU scan.

Both FDG scans were acquired and images were reconstructed using local procedures, but it was mandated that each of the scans within the same patient was processed with exactly the same protocol and specifications (same scanner, UT, scanner setting for acquisition and reconstruction, axial body coverage area). Both 2D and 3D acquisitions were allowed, as long as the technique was identical for both scans.

### Imaging sites and scanner calibration

Eleven sites agreed to participate in this sub-study, all large academic institutions. Inter-institutional variations in SUV due to scanner and protocol differences were expected. Therefore, strict adherence to standardized acquisition and processing protocols was required together with calibration of the PET and PET/CT scanners used in this study prior to first patient enrolment.

In order to verify image quality, consistency of acquisition and to ensure sites could export the image data to a central data storage, upload of an anonymized test data set to a central server was requested prior to recruitment of patients.

It was specified in the IG that all scans had to be submitted in standard DICOM format for central data storage within 48 h of acquisition. An ITF had to be submitted along with every scan. The ITFs and DICOM headers were analysed retrospectively for compliance with the requirements indicated in the IG. Proper image anonymization was verified as well, i.e. replacing patient name and hospital ID with the provided sequential ID for the study.

### Assessment of the scans

- Visual scoring for quality of the scans was planned using a three-point scale (good: absence of artefacts, good signal to noise; intermediate: absence of artefacts and reasonable signal to noise; poor: presence of artefacts and very low signal to noise) by consensus from three expert readers.
- For the lesions identified for RECIST on the diagnostic CT scans stipulated in the main protocol, SUV were measured in the region of interest of each lesion and were normalized for body weight, but uncorrected for blood glucose values. SUV_max_, being defined as the maximum single voxel SUV within the region of interest, was used for the quantification of the lesions and had to be measured on the same tumour lesions at BL and during therapy.
- For QC of the FDG distribution, a volume of interest (VOI, range 12.34–14.7 cm^3^) was drawn on the PET images in an area of normal liver tissue containing homogeneous FDG distribution. The mean liver standardized uptake value (LSUV_mean_) was used as an additional QC parameter as it becomes more and more accepted in the nuclear medicine community. It is an indirect and combined measure of scanner calibration, net injected dose, proper administration and clock synchronization. The LSUV_mean_ in a 3D VOI can be expected to be well within the 1–3 range [2].

## Results

### Patient recruitment and scan collection

Enrolment of 50 patients into the PET sub-study was planned. However, since the main trial accrued very rapidly while the IG were developed at a rather late stage, only 44 patients could actually be enroled which should have resulted in 88 scans (1 BL and 1 FU scan per patient). Of 88 scans, 81 were uploaded to the central database. In two patients there was no definitely metabolically active lesion at BL so no FU scan was performed; two patients were never included in the PET imaging protocol as they did not meet the screening criteria for the imaging sub-study; two patients were taken off the study before the FU FDG scan was performed and in one patient the BL scan could not be retrieved by the site.

Of the 81 scans received, proper anonymization had been performed on 36 scans by the sites submitting the scans. This includes at least replacement of the patient name and hospital ID with the respective enrolment subject number in all DICOM images submitted.

### Imaging sites, scanner calibration and test data

General site visits were performed, but not specific to the FDG imaging. Due to a more rapid patient accrual than foreseen in the main study, late integration of this FDG sub-study and lack of resources, scanner calibration before enrolment of patients proved to be unfeasible, nor was the collection of individual phantom scans for review of reconstruction parameters and image quality evaluation under specified conditions. Of 11 sites, 6 submitted test data prior to patient accrual; the other sites denominated their first patient’s scan as the test data scan. Retrospective review of DICOM headers in the six test data scans revealed that in three data sets the UT was outside the specifications in the IG.

### Serum glucose compliance

According to the IG, serum glucose values should have been submitted for each of the 81 scans and determined prior to each scan. Of 81 serum glucose values, 77 were submitted, all but 1 being within the limits set out in the guidelines: average 97 mg/dl (5.4 mmol/l), median 94 mg/dl (5.2 mmol/l), range 61–200 mg/dl (3.4–11.1 mmol/l). At FU, one patient with serum glucose of 200 mg/dl was reported as diabetic, but not indicated on the ITF for the BL scan.

### Visual quality assessment

**Fig. 1:**
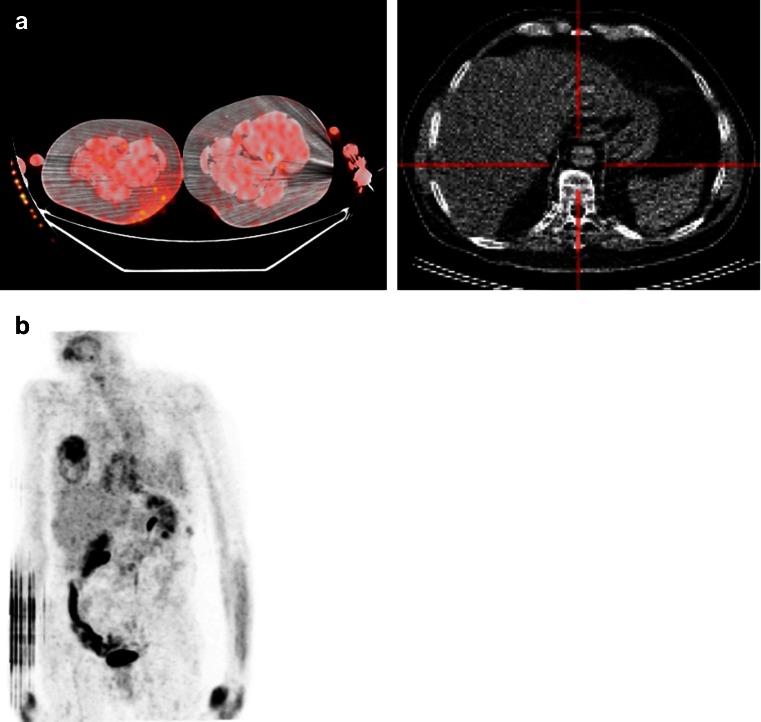
Representative artefacts

A retrospective review and visual quality assessment was performed on all 81 scans received by three nuclear medicine physicians by consensus using OsiriX (www.osirix-viewer.com) software. In 35/44 patients both scans were received and of sufficient quality to perform visual assessment. The quality of the BL scan was good in 88.6 % of patients and intermediate in 11.4 %. The same results were observed for the quality of the FU scans. Figure 1 includes representative artefacts observed during the visual assessment.

### FDG UT compliance

The UT is a major factor affecting SUV [9]. It was observed that the UT in the DICOM headers in some instances was different from the information provided on the ITF, often outside of the prescribed 60 ± 5 min as per the IG. For these reasons, a complete review of both scans was done in the 35 visually assessable patients. The injection and scan start time from the DICOM headers (using the OsiriX software) and ITF were recorded and compared. In 22/70 ITFs, the UT differed by more than 1 min from the UT in the DICOM headers; the maximum difference was 1 h 4 min.

**Fig. 2:**
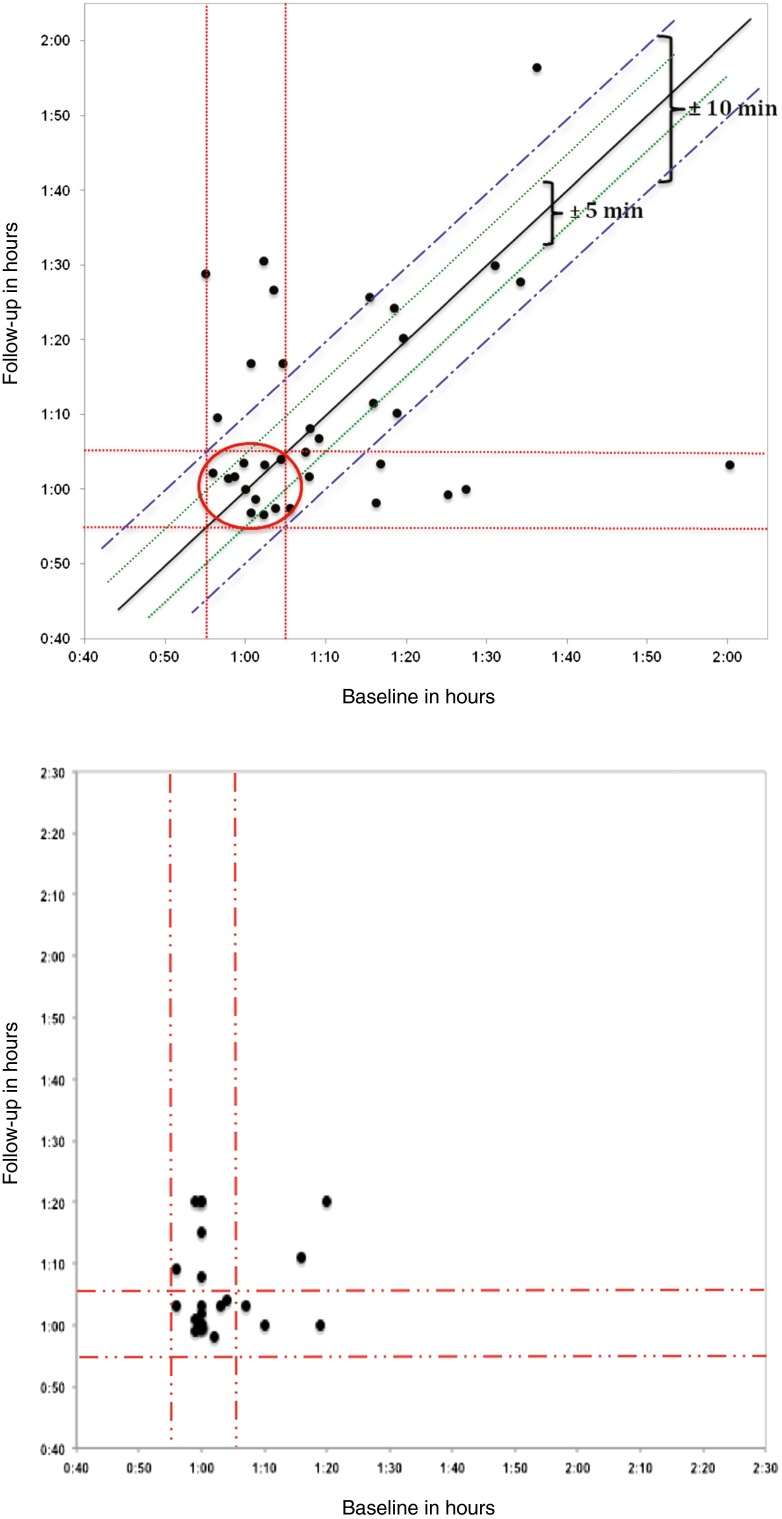
**a** UT between FDG injection and start of the PET scan, based on DICOM tags **b** UT between FDG injection and start of the PET scan, based on ITFs

We considered the UT retrieved from the DICOM headers reliable and representative for the actual UT (Fig. 2a) compared to the UT marked on the ITF (Fig. 2b). Compliance with the IG with regard to UT for both scans was 31.4 % (11/35 patients). The actual BL UT ± 10 min at FU resulted in 66 % compliance (23/35). However, due to the uncontrolled diabetes in 1 patient, 22/35 patients were considered for semi-quantitative assessment using SUV_max_. Furthermore, due to technical problems with the PET system, FU scans for two patients were acquired on different scanners.

### Consistent scanning between BL and FU

- Same scanner

In 2/35 patients different PET/CT systems were used for the BL and the FU scans due to scanner breakdown (Table 1). Information on the reconstruction method to confirm consistency was not available.

**Table 1:** Reconstruction parameters

Site code	PET slice thickness (mm)	Scan direction	Scanner	FDG dose (MBq)	Time per bed (s)	Reconstruction method
A	2	Cranial	Siemens Biograph 16	272–378	180	OSEM2D 5i8s
B	4	Cranial	Philips Allegro Body©	312–400	180	3D-RAMLA
B	4	Cranial	Philips GEMINI TF TOF 16	192–195	120	3D-RAMLA
C	3.27	Cranial	GE Discovery ST	221–258	180	3D FORE IR
D	5	Cranial	Philips GEMINI TF©	191	90	BLOB-OS-TF
D	5	Cranial	Philips GEMINI TF TOF 16	164–238	60–90	BLOB-OS-TF
E	3.38	Cranial	Siemens Biograph Duo	202–370	240	OSEM2D 2i8s
F	4	Cranial	Philips Allegro Body(C)	349–369	120	3D-RAMLA
F	4	Cranial	Philips GEMINI TF©	349–371	120	BLOB-OS-TF
F	4	Cranial	Philips GEMINI TF TOF 16	369–407	120	BLOB-OS-TF
G	2.43	Cranial	Siemens Biograph Duo	275–343	210	OSEM 2i8s
H	3.27	Cranial	GE Discovery STE	379	150	3D IR
I	4.25	Cranial	GE Discovery LS	323–381	86–240	OSEM
I	4.25	Caudal	GE Advance	331–381	240	Unknown
J	4	Caudal/cranial	Philips GEMINI TF©	235–291	120	BLOB-OS-TF
K	2	Cranial	Siemens Biograph 16	706–812	240	OSEM2D 4i8s
K	3.27	Cranial	GE Discovery ST	645–813	300	OSEM

- Scan direction

Keeping scan direction consistent between a BL and all FU scans is paramount. In 2/35 patients the scan direction was changed between BL and FU and in 2/35 the scan direction could not be determined based on the DICOM tags’ information most likely due to either anonymization or scanner standard of functionality (Table 1).

- Time per bed

In 3/35 patients there was minor change in time per bed between BL and FU (≤4 s), and in 1/35 the difference was 50 % increase from BL to FU (Table 1). For the latter case, dose change from BL to FU was 18.8 % decrease, while no patient weight change was reported.

All confounding factors together (scan visual quality, deviations in UT, deviations in scanning protocol, scanner change) resulted in an ultimate sample size too small to perform the quantitative analysis for prognostic and predictive factors.

### LSUV_mean_

The LSUV_mean_ was measured via VOI in 34/35 patients (68 scans), whose scans were considered visually acceptable. In 1/35 subjects normal liver uptake could not be reliably identified due to extensive hepatic tumour deposits.

Average and range of LSUV_mean_ data were assessed for scans falling into three ‘qualitative’ categories:

- UT according to the IG for both FDG scans 60 ± 5 min (11 patients)
- UT of FU FDG scan within the actual BL UT ± 10 min, irrespective of the absolute UT of the BL scan (12 patients)
- UT outside the specifications of the IG and more than 10 min difference between BL and FU scan (11 patients)

**Fig. 3:**
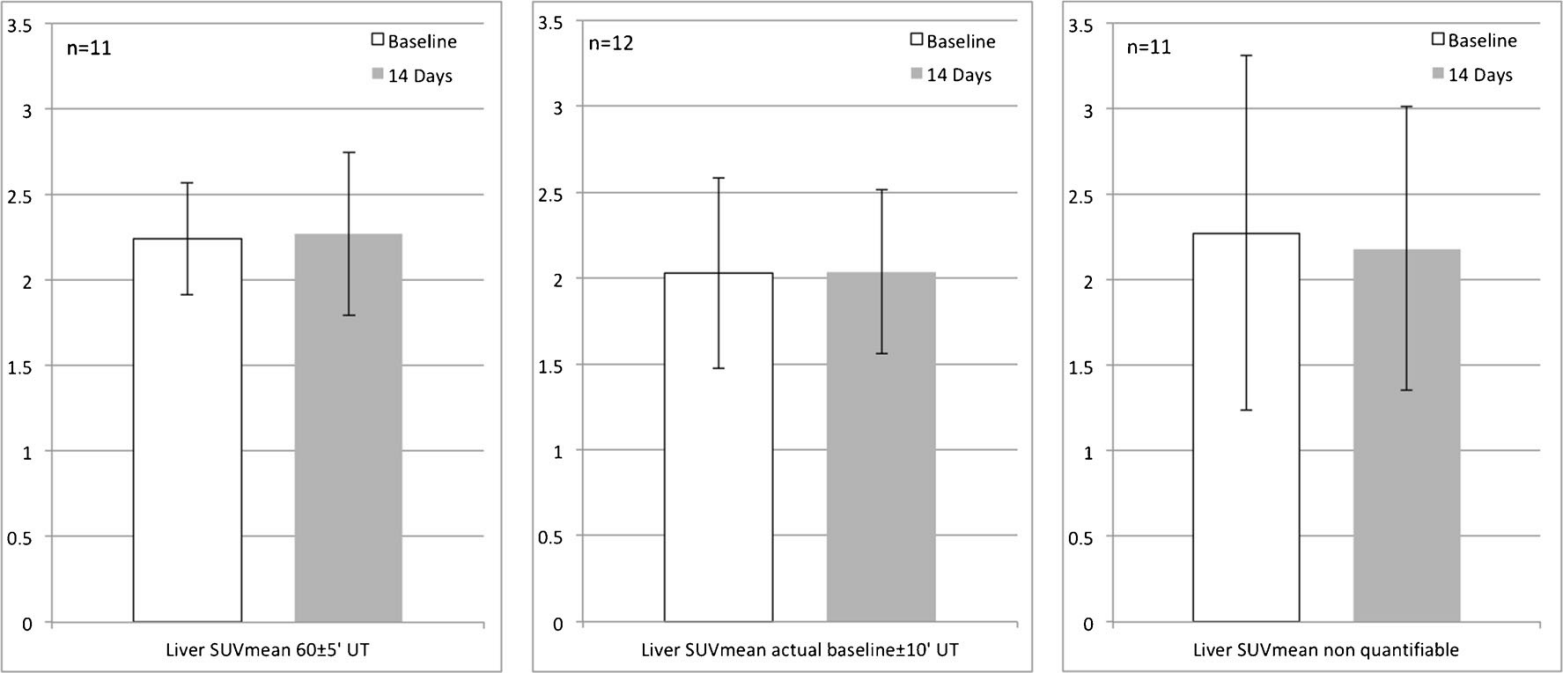
Liver SUV_mean_ in normal liver tissue **a** UT was according to the IG for both FDG scans: 60 ± 5 min (11 patients). **b** UT of FU FDG scan was within the UT of the actual BL scan ± 10 min, irrespective of the absolute UT of the actual BL scan (12 patients). **c** UT outside the specifications of the IG and more than 10 min difference between BL and FU scan (11 patients)

As shown in Fig. 3, LSUV_mean_ was fairly constant among the 11 patients with excellent UT compliance and very low SD of the LSUV_mean_: 2.24 ± 0.33 at BL and 2.27 ± 0.48 at FU. Variability in SD of LSUV_mean_ substantially increased for the subjects with UT outside the acceptable range (11 patients): 2.27 ± 1.04 at BL and 2.18 ± 0.83 at FU.

## Discussion

The variability in local standard of care in the use of PET/CT across centres has been well documented [10, 11]. The results from this multicentre international FDG sub-study confirm that this has a large impact on data with FDG scans used for primary endpoint. Providing IG to the sites is not sufficient. While retrospective QC is useful to identify compliant and noncompliant data, the results from this study clearly demonstrate that prospective QC is of the utmost importance as it has an impact on study outcome [12]. In retrospect, it is almost impossible to retrieve the necessary data or to have any impact on improving quality. As documented by Velasquez et al. [13], centralized QA in the multicentre multiobserver settings greatly improves the final outcome. The results presented here confirm that prospective rapid QC review and feedback is mandatory to improve compliance with the IG, and is imperative to ensure that imaging data used for endpoints are in fact reliable, rather than a mere reflection of change in imaging technique used. Besides general site visits by monitors, specific site visits with respect to the imaging scans would improve both compliance and quality.

Some sources of error in PET quantification can be readily identified. Unfortunately, it is an inevitable shortcoming that the injection time in the DICOM header is stored by manual input in the scanner computer, and these data cannot be stored appropriately in the DICOM header. As it is impossible to separately store information on the injection time and the calibration time, the operator has to choose, resulting in one missing time point. Furthermore, sponsors rely on the sites to ensure synchronization of all clocks in the PET department. It is felt that instances of erroneous input of injection time and non-synchronization of clocks reflect the real world situation, but are rather very infrequent. Studies have shown the use of LSUV_mean_ as a reliable marker for QC purposes of scans longitudinally [14]. The data presented here confirm that compliance with the IG has a significant impact on this parameter, and it should be considered as a metric during QC of FDG scans.

Design and execution of a multicentre international imaging sub-study as part of a larger clinical study (i.e. translational research study) requires extensive planning with many considerations. Proper power calculation of the study should include certain excess attrition rate due to poor quality of scans, patient dropout or ineligibility based on BL scan. At the time of this project, no reliable data in the literature were available to suggest what could be a reasonable number of attrition for the above-noted reasons. Proper on-site training of all involved staff and prospective QC with rapid feedback to sites could improve compliance and quality of the collected imaging data and are prerequisite to obtain data sets with sufficient statistical power for making study-related conclusions.

Poor compliance with the study IG and inadequate quality of scans, when major artefacts are observed affecting visual and quantitative assessment, should be identified and reported to the sites and the sponsor immediately prior to the FU scan or the next patient enrolment. Mandatory test data submission and review, prompt feedback to sites and site authorization pending test data approval have proven very important, leading to increased and sustainable compliance with the imaging protocol [12, 15]. The recently proposed approach by van den Hoff et al. [16], allowing comparison of data sets from different acquisition times, may correct for differences in UT. However, it may not be performed with the same accuracy on other data sets or may need some data set-specific recalibration. In general, avoiding the need for corrections or at least minimizing the potential impact of any correction should be preferred. Thus, performing the studies with standardized imaging procedures should be the aim.

To reach an international standardization and harmonization in the use of QIBs in multicentre clinical studies, producing high-quality reliable results, it is paramount to ensure prospective QC in the execution of the imaging protocol and obligatory compliance with the specified parameters. It was not until the second half of 2011 that the US Food and Drug Administration (FDA) published central review charter draft guidelines [17] for the industry, addressing both challenges in part. In clinical studies where efficacy is assessed with the aid of QIBs, the imaging protocol should become a regulatory document and complied with in the same regard as the actual clinical study protocol. The sponsor has an obligation to ensure that the study protocol mandates strict compliance with the IG and a plan for protocol violations should be put in place.

Molecular imaging with QIBs could have a major impact benefiting patients (earlier start of alternative treatment or spare of side effects in case of ineffective treatment) as well as the health care system (lower financial burden associated with ineffective treatment). For clinical studies, this may lead to a shorter duration of trials, improved cost-effectiveness and faster approval of compounds by the regulatory agencies. Pay for performance or P4P is a programme initiated in the UK [18] (in 2004) and the USA (in 2005) aiming to ensure high-quality health care delivered to patients is rewarded. In 2008 the Medicare Improvements for Patients and Providers Act [19] (MIPPA) was accepted. It applies to physicians, non-physician practitioners and independent diagnostic testing facilities as suppliers of imaging procedures who are submitting claims for the technical component of advanced diagnostic imaging procedures. All such suppliers in order to receive reimbursement from the Centers for Medicare and Medicaid Services (CMS) are obligated to obtain and maintain accreditation of their imaging facilities. However, PET/CT scanners used for imaging patients in multicentre clinical studies require further harmonization. At the time this FDG sub-study design was finalized (2008), no formal programme for cross-calibration of scanners existed. Since then, the SNM Clinical Trials Network (CTN) [20], American College of Radiology (ACR) [21] and EANM Research Ltd (EARL) [22] have developed programmes for PET/CT scanners’ harmonization and qualification to participate in multicentre clinical studies. However, a requirement by formal government or regulatory agencies to mandate the use of those programmes has not yet been proposed. It is in the best interest of the public, the academia and the pharmaceutical industry to use the accreditation programmes effectively and rigorously, ensuring data comparability across sites and longitudinal patient’s scans.

Standardized imaging protocols for FDG scans have been published by Shankar et al. [9] and Boellaard et al. [23] and are available for implementation within multicentre clinical studies. A more recent publication in the process of finalization is the effort of the QIBA Profile: FDG PET/CT as an Imaging Biomarker Measuring Response to Cancer Therapy with the Uniform Protocols in Clinical Trials (UPICT) [24]. Yet from a recent publication by Rausch et al. [25] it is clear that day-to-day practice is far from standardized in a single country.

Imaging sub-studies should be developed in parallel with the main study to ensure readability of the translational research project at the same time as the main study. A recent publication from Josephson et al. [26] discussed many of the barriers in front of drug development as to the use of QIBs in translational sub-studies. In addition, a very basic factor such as rapid recruitment in the main study, later start of the imaging sub-study and inability to recruit enough patients for the latter must be seriously considered. The sponsor must foresee possible inability to achieve the preplanned statistical design, leading to an underpowered sub-study, or even the main study (if QIBs are used for the endpoints and compliance is poor), which could also be due to low compliance and quality of the scans.

Great effort has been undertaken by the SNM and EANM towards the importance of understanding International Conference on Harmonization-Good Clinical Practice (ICH-GCP) guidelines and ensuring that the imaging department staff is trained in GCP. Yet little to no support has been given to this goal from the pharmaceutical industry or recruiting investigators at sites. Increased GCP education for the imaging personnel involved in the chain of patients’ scans from the ordering of scans to the delivery of the imaging data to the sponsor will improve the results. If a patient is referred for a PET scan, yet the imaging department is not informed that the patient is taking part in a specific clinical study, requiring the use of an imaging protocol different from the local routine clinical practice, this could hamper compliance and result in a high attrition rate of data for the endpoint of the sub-study. The results from this review indicate that low compliance with the IG warrants intense education on the basic GCP principles such following the prescribed IG and properly de-identifying the data submitted to the sponsor.

Validation of FDG scans for early treatment response and patient stratification has great potential, but is pending due to conflicting results being presented in the literature, while no data on IG compliance are required to be published by the investigators and the sponsors as of yet.

## Conclusion

The use of QIBs in patient care and novel therapies is feasible, but only when all involved in the clinical study jointly aim at achieving high quality. A QIB such as FDG is a very sensitive marker to IG deviations, requiring strict adherence to protocol. Rigorous compliance with the IG is as crucial as compliance with the clinical protocol, as shown in this study. IG noncompliance with respect to UT resulted in a large variability in LSUV_mean_ and could possibly affect tumour uptake quantification. Effective approaches in the use of QIBs have been proposed by various academic groups and scientific societies. Regulatory authorities, the pharmaceutical industry (including imaging companies) and investigators play a pivotal role in developing and implementing standardized processes for the use of QIBs. Increased education of the imaging personnel in multicentre clinical trials will improve compliance with the IG and ultimately deliver better cost-effective surrogate endpoints of clinical studies. Government efforts towards laws and rules mandating the use of accredited QIB facilities in multicentre studies could facilitate the desired change in the quality of the results.
